# Segmental and total uniparental isodisomy (UPiD) as a disease mechanism in autosomal recessive lysosomal disorders: evidence from SNP arrays

**DOI:** 10.1038/s41431-019-0348-y

**Published:** 2019-02-08

**Authors:** Ineke Labrijn-Marks, Galhana M. Somers-Bolman, Stijn L. M. In ’t Groen, Marianne Hoogeveen-Westerveld, Marian A. Kroos, Sirpa Ala-Mello, Olga Amaral, Clara sa Miranda, Irene Mavridou, Helen Michelakakis, Karin Naess, Frans W. Verheijen, Lies H. Hoefsloot, Trijnie Dijkhuizen, Marloes Benjamins, Hannerieke J. M. van den Hout, Ans T. van der Ploeg, W. W. M. Pim Pijnappel, Jasper J. Saris, Dicky J. Halley

**Affiliations:** 1 Department of Clinical Genetics, Erasmus University Medical Center, Rotterdam, The Netherlands; 2 Molecular Stem Cell Biology, Department of Clinical Genetics, Erasmus University Medical Center, Rotterdam, The Netherlands; 3 Department of Pediatrics, Division of Metabolic Diseases and Genetics, Erasmus University Medical Center-Sophia, Rotterdam, The Netherlands; 4 Center for Lysosomal and Metabolic Diseases, Erasmus University Medical Center, Rotterdam, The Netherlands; 5 Department of Clinical Genetics, Helsinki University Hospital, Helsinki, Finland; 6 Department of Human Genetics, Unit of Research and Development, National Institute of Health Dr Ricardo Jorge, Porto, Portugal; 7 Unilipe, IBMC-University of Porto, Porto, Portugal; 8 Department of Enzymology and Cellular Function, Institute of Child Health, Athens, Greece; 9 Centre for Inherited Metabolic Diseases, Karolinska University Hospital, Stockholm, Sweden; 10 Department of Genetics, University Medical Center Groningen (UMCG), Groningen, the Netherlands

**Keywords:** Genetic testing, Genetic testing, Medical genetics, Disease genetics, Laboratory techniques and procedures

## Abstract

Analyses in our diagnostic DNA laboratory include genes involved in autosomal recessive (AR) lysosomal storage disorders such as glycogenosis type II (Pompe disease) and mucopolysaccharidosis type I (MPSI, Hurler disease). We encountered 4 cases with apparent homozygosity for a disease-causing sequence variant that could be traced to one parent only. In addition, in a young child with cardiomyopathy, in the absence of other symptoms, a diagnosis of Pompe disease was considered. Remarkably, he presented with different enzymatic and genotypic features between leukocytes and skin fibroblasts. All cases were examined with microsatellite markers and SNP genotyping arrays. We identified one case of total uniparental disomy (UPD) of chromosome 17 leading to Pompe disease and three cases of segmental uniparental isodisomy (UPiD) causing Hurler-(4p) or Pompe disease (17q). One Pompe patient with unusual combinations of features was shown to have a mosaic segmental UPiD of chromosome 17q. The chromosome 17 UPD cases amount to 11% of our diagnostic cohort of homozygous Pompe patients (plus one case of pseudoheterozygosity) where segregation analysis was possible. We conclude that inclusion of parental DNA is mandatory for reliable DNA diagnostics. Mild or unusual phenotypes of AR diseases should alert physicians to the possibility of mosaic segmental UPiD. SNP genotyping arrays are used in diagnostic workup of patients with developmental delay. Our results show that even small Regions of Homozygosity that include telomeric areas are worth reporting, regardless of the imprinting status of the chromosome, as they might indicate segmental UPiD.

## Introduction

Up to date diagnostics of metabolic disorders includes mutation analysis of the causative gene. Although enzyme assays have been considered to be the gold standard for diagnosis of the symptomatic patient, genotyping is a requirement for inclusion in enzyme replacement programs and is a prerequisite for carrier tests in relatives and DNA-based prenatal diagnosis. For confirmation of allelic segregation, we routinely request parental DNA samples. In this report, we will refer to those sequence variants known to cause (Hurler or Pompe) disease in a homozygous or compound heterozygous state as “pathogenic”.

In recent years, we encountered four cases of apparent homozygosity for pathogenic variants that could not be confirmed by parental DNA analyses, as only one of the parents was a heterozygote. The first was a child with mucopolysaccharidosis type I (Hurler disease, OMIM 607014); three other patients were afflicted with glycogenosis type II (Pompe disease, OMIM 232300). These four cases represented 53 homozygous patients with either Hurler- or Pompe disease where the availability of parental DNAs allowed segregation analysis.

Since (partial) gene deletions can generate false homozygosity the relevant gene (*IDUA* and *GAA*) regions of the first two patients were interrogated by comparative real time (quantitative) qPCR. Also, we searched for SNPs in the amplicons’ primer regions per resequencing.

A special diagnostic case was a young child with cardiomyopathy (but no muscle weakness), possibly related to Pompe disease, who was found to have decreased - but not deficient - acid α-glucosidase activity in leukocytes, but a deficiency in cultured fibroblasts. Onset of cardiomyopathy within the first year without skeletal muscle weakness, with a sizable (residual) activity of acid α-glucosidase in leukocytes and fibroblasts, represents an uncommon combination of features when compared with descriptions of the clinical spectrum of Pompe disease [1, 2]. His *GAA* genotype showed apparent *heterozygosity* for a paternally inherited pathogenic sequence variant, with some skewing due to an enhanced paternal contribution and different patterns in leukocytes and fibroblasts.

Uniparental disomy (UPD) is the inheritance of both homologues of a pair of chromosomes from one parent only. Heterodisomy means that both parental homologues are present, while isodisomy refers to the presence of two copies of one parental homologue. Segmental UPD is defined as UPD of a part of one chromosome together with biparental inheritance of the rest of this pair of chromosomes. Problems resulting from UPD are aberrant genomic imprinting and, in the case of isodisomy, of “homozygosity of autosomal recessively inherited mutations” [3].

Subsequent analyses of these five cases with microsatellite markers and SNP genotyping arrays showed uniparental (iso)disomies. We report four cases of *segmental* UPiD and show that a *mosaic* form of sUPiD can generate a phenotype with unusual combinations of features. UPiD may be an underestimated cause of autosomal recessive disease. Our results bear relevance to genetic counseling of families with autosomal recessive diseases as well as diagnostics of patients with mild or unusual phenotypes and diagnostic workup of patients with developmental disorders of unknown aetiology using modern screening techniques.

## Materials and methods

### Patients

For patient A, a clinical diagnosis of mucopolysaccharidosis type I or type VI was considered at the age of 11 months. Arylsulfatase B activity in leukocytes was in the normal range, whereas α-L-iduronidase showed a profound deficiency (0.2 nmoles/17 h/mg, with a normal range of 25–90). This established the diagnosis of mucopolysaccharidosis type I (Hurler disease). He was on enzyme replacement therapy at the age of 13 months and received an allogenic stem cell transplant at 1 year and 4 months. He has been free of infections after splenectomy. At 12 years of age, his cognitive status was at the level of 8 years.

Patient B was born from a consanguineous marriage. Her parents are first cousins. The diagnosis of childhood Pompe disease was established by the demonstration of deficiency of acid α-glucosidase in cultured fibroblasts when the patient was 3 years old (0.24 nmoles/h/mg using 4MU substrate with a normal range of 40–180). Since the parents opted for PGD (preimplantation genetic diagnosis) in a subsequent pregnancy mutation analysis of the *GAA* gene was requested.

Patient C was hospitalised with respiratory distress 6 weeks and 5 days after birth. The diagnosis of Pompe disease was established by the demonstration of deficiency of acid α-glucosidase in cultured fibroblasts (0.1 nmoles/min/mg using 4MU substrate with a normal range of 0.34–2.4 and a patient range of 0.01–0.3). The patient developed cardiomyopathy and hepatosplenomegaly. Myozyme enzyme replacement therapy was started 11 days after admission to the hospital, but his condition deteriorated and the infant died with cardiorespiratory arrest 3 weeks after admission.

Patient D, a child of mixed Scandinavian ethnicity, was diagnosed with Pompe disease at 16 months of age. His diagnosis was based on clinical symptoms, including hypertrophic cardiomyopathy and generalized muscle weakness, and acid α-glucosidase deficiency, measured in isolated lymphocytes. He was treated with Myozyme from the age of 17 months. DNA was received by our laboratory (Clinical Genetics, Rotterdam) for mutation analysis when he was two years of age. There were urgent clinical reasons to start enzyme replacement therapy immediately after the biochemical diagnosis had been made and ahead of genotyping. Also, the clinical and enzymatic features of the patient made genetic null variants, which would have been a contraindication, unlikely; the patient’s positive cross-reactive immunological material status supported this decision.

Patient E was born after an uneventful pregnancy. On a prenatal ultrasound a persistent left superior vena cava was suspected. Therefore he had a cardiac ultrasound at the age of 2.5 months. A left ventricular hypertrophy was found, which later developed into a biventricular cardiac hypertrophy. Motor development was normal and the child had normal strength. As a toddler he does not show symptoms of muscle weakness or any other clinical symptoms; this makes him a very unusual case. Because of the infantile cardiomyopathy a diagnosis of Pompe disease was considered. The acid α-glucosidase activity in leukocytes was decreased (26 nmoles/h/mg) but clearly above the activity in known Pompe patients (range normal controls 40–250, patients 0–10). However, in cultured fibroblasts, the activity was 8.1 nmoles/h/mg using 4MU substrate (with a normal range of 45–180 and a patient range of 0–20).

### DNA isolation and mutation analysis

DNA was extracted from blood using Magnetic Separation Module 1 from Chemagen (Baesweiler, Germany). Routine mutation analysis of the *IDUA* and *GAA* genes was done by sequencing of the coding exons and intron exon boundaries, i.e. exons 1–14 (*IDUA*; NM_000203.3 (GRCh37/hg19)), exon numbering as in https://databases.lovd.nl/shared/refseq/IDUA_NM_000203.3_table.html and Kwak et al. [4], and 2–20 (*GAA*; NM_000152.3 (GRCh37/hg19)), exon numbering as in https://databases.lovd.nl/shared/refseq/GAA_NM_000152.3_table.html and Kroos et al [5]. (In addition, the (relatively frequent) deletion of exon 18 of *GAA* was tested with a separate deletion specific PCR.) Primers were designed using standard software (Primer3, http://frodo.wi.mit.edu/cgi-bin/primer3/primer3_www.cgi); sequences available on request. PCR products were purified with ExoSap-it (USB, Cleveland, Ohio, USA) and sequenced using the Big Dye Terminator kit according to the supplier’s instructions (Applied Biosystems, Foster City, California, USA). Sequence reaction products were purified with Performa DTR V3–96 well short plates (Edge BioSystems, Gaithersburg, Maryland, USA) and analysed using an ABI 3730 XL analyzer (Applied Biosystems) and SeqScape (Applied Biosystems) or SeqPilot software (JSI medical systems GmbH).

In vitro functional testing of a *GAA* missense variant using site directed mutagenesis was performed as published by Kroos et al. [5]. Data on the variants and the patients were submitted to www.lovd.nl/IDUA and www.lovd.nl/GAA. Patients A-E were registered as #00183080, #00183081, #00183082, #00183083 and #00183084, respectively.

### Identity testing

The AmpFlSTR® Identifiler® PCR Amplification Kit was used to verify the origin of patients’ and parental samples.

### qPCR analysis - Copy number variation detection

Two real-time PCR techniques were used to detect a possible deletion in *IDUA* and *GAA* (http://www.eurogentec.com/8233Discover+our+new+qPCR+guide.html). First, TaqMan Probe assays were designed using Primer Express 2.0 (Applied Biosystems) and performed on an ABI 7500, using qPCR low ROX MasterMix Plus (Eurogentec). For *IDUA* exons 1–14 were tested and for the *GAA* gene exon 3, 8, 13 and 18. Subsequently, results were verified with SYBR green assays on an ABI 7900 using MESA GREEN qPCR MasterMix Plus for SYBR assay Low Rox (Eurogentec) for exon 2 of the *IDUA* gene and exon 13 of the *GAA* gene using the TaqMan assay outer primers.

### Marker analysis

Microsatellite markers for chromosome 4 (*n* = 8) and chromosome 17 (*n* = 15) were selected from the GB AB Map (ABI PRISM Linkage Mapping Set Version 2, Panel Guide and the UCSC hg19 genome browser (http://genome.ucsc.edu/cgi-bin/hgGateway). The VIC or FAM labelled PCR products were run on the ABI 3730 XL. The markers are summarised in Table 1.

**Table 1:** Markers on chromosome 4 and 17 used for this study

Marker	Chromosome	Start pos. (hg19)	End pos. (hg19)
D4S412/a	4	3380692	3381012
D4S2935/b	4	6560881	6561231
D4S403/c	4	13750828	13751166
D4S405/d	4	40352512	40352915
D4S1592/e	4	57681811	57682157
D4S1572/f	4	103769921	103770290
D4S415/g	4	178711416	178711101
D4S1535/h	4	185235750	185236098
D17S831/i	17	1910400	1910767
D17S1852/j	17	10515501	10515829
D17S921/k	17	14260599	14260928
D17S1857/l	17	16415217	16415593
D17S1824/m	17	26659952	26660227
D17S798/n	17	31289812	31290190
D17S927/o	17	35006344	35006731
D17S1868/p	17	47184746	47185087
D17S944/q	17	61436174	61436545
D17S785/r	17	74431300	74431581
D17S1790/s	17	75297293	75297627
D17S802/t	17	76234526	76234797
D17S836/u	17	77299839	77300058
D17S784/v	17	77802121	77802424
D17S928/w	17	80252839	80253140

/a etc refers to the position in Fig. 2.

### SNP microarray analysis

For patients A and B, SNP array analysis was performed with Affymetrix SNP6.0 arrays according to the manufacturer’s protocol (Santa Clara, CA, USA) as described [6]. (Affymetrix is now part of ThermoFisher Scientific, Waltham, MA, USA). DNA trio analysis of patient C and parents was done on the 300 K Illumina (San Diego, CA, USA) HumanCytoSNP-12 BeadChip platform [7, 8] and trioanalyses of patients D and E and their parents were done on the Illumina InfiniumCytoSNP-850K BeadChip platform [8, 9]. These platforms had (consecutively) become part of our standard clinical laboratory procedure. A cascade of algorithms was applied for copy number analysis and genotyping, including Genome Studio (Illumina), GTC (Affymetrix) and Nexus CopyNumber™ (Biodiscovery, El Segundo, CA, USA). Analysis of LOH (Loss of Heterozygosity) was based on B-allele frequency calculation (BAF), the B-allele representing the minor non-reference allele. Expected values for BAF are 0 for AA, 0.5 for AB and 1 for BB, meaning ∆BAF represents estimated deviation from the expected AB value.

## Results

### Mutation analysis of patients and parents

**Fig. 1:**
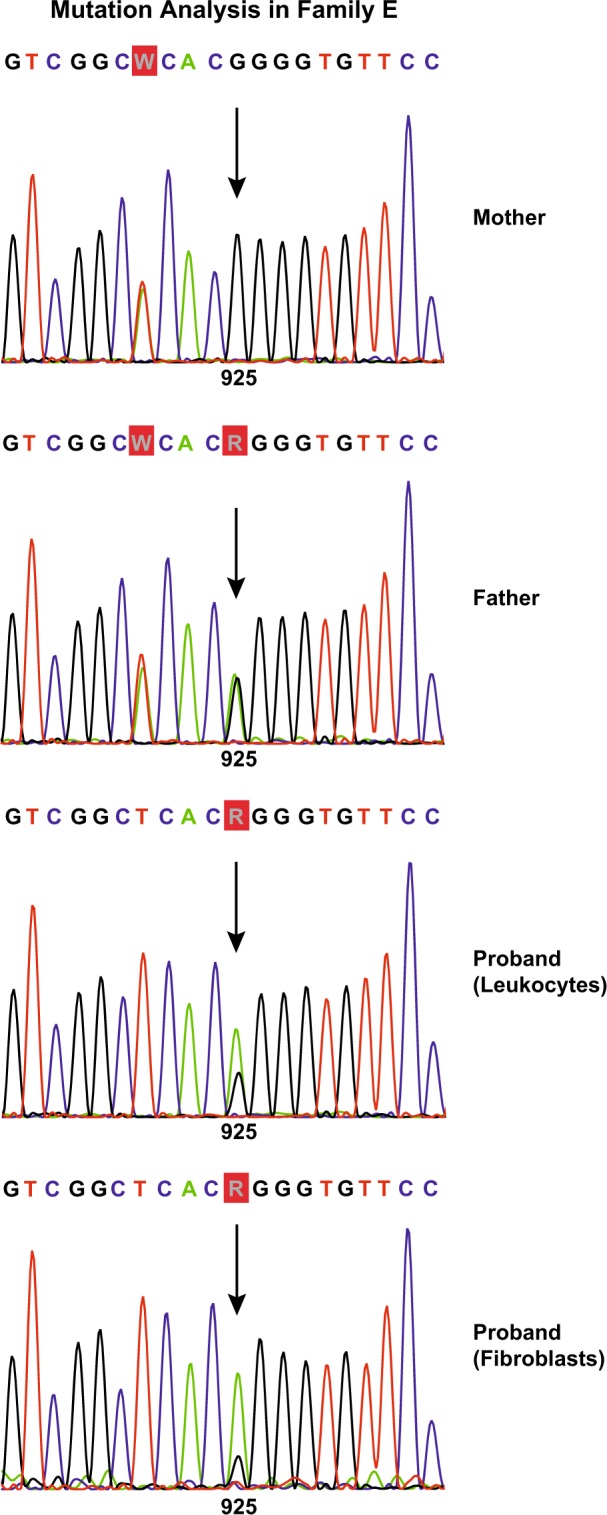
Sequence analysis of exon 5 of the *GAA* gene. Both parents are heterozygous for c.921A > T, a silent change (p.Ala307 = ), the proband shows a homozygous T pattern. Position 925 is indicated by arrows. The father is heterozygous for c.925G > A; p.(Gly309Arg), a known pathogenic variant, mother has wild type sequence. The proband shows skewed, pseudoheterozygous, patterns in leukocytes and (briefly) cultured fibroblasts, with the highest mutant peak in the fibroblasts

After DNA sequencing, the Hurler disease patient A, presented as homozygous for the founder (nonsense) variant c.208 C > T, p.(Gln70*) in exon 2 of the *IDUA* gene (chromosome 4p16.3) known to occur in patients with Hurler disease in a homozygous or compound heterozygous state. Subsequently, both parents were tested and the mother was heterozygous for c.208 C > T, p.(Gln70*); the father, however, tested negative. Pompe patient B appeared to be homozygous for the novel nonsense variant c.1853G > A, p.(Trp618*) in exon 13 of the *GAA* gene (chromosome 17q25.2-q25.3). Both parents were tested, with mother heterozygous and father negative for c.1853G > A, p.(Trp618*). In both families paternity was confirmed. Repeated sequencing of the patients’ DNA using new sets of primers outside the originally amplified fragments did not reveal any SNPs that might have compromised the detection of the paternal allele in the previous tests. Two more patients were encountered, both with sequence alterations in exon 5 of the *GAA* gene: Pompe patient C, with apparent homozygosity for the founder pathogenic missense variant c.925 G > A, p.(Gly309Arg), and Pompe patient D, whose DNA showed a homozygous pattern for the novel missense change c.871 C > T, p.(Leu291Phe), of which the clinical relevance was unknown. The functional significance of this change was studied using site directed mutagenesis. The resulting classification was a ‘pathogenic potentially severe to slightly less severe’ variant. Also in these cases only the patients’ mothers carried the variant. Paternity was confirmed. Patient E, who had hypertrophic cardiomyopathy, but no muscle weakness, had decreased (but not deficient) acid α-glucosidase activity in leukocytes, but a deficiency in cultured fibroblasts. *GAA* genotyping was done on DNA from leukocytes and from fibroblasts. In both DNA samples a pattern with a normal (maternal) and a mutant (paternal) allele was observed. It concerned the founder pathogenic missense variant c.925 G > A, p.(Gly309Arg). Each of the patterns was skewed, in the sense that the (mutant) A peak was higher than the G (Fig. 1), which was more pronounced in the fibroblast DNA.

### Comparative qPCR with TaqMan Probe and SYBR green assays for copy number quantification

We continued the analyses of patients A and B using qPCR with TaqMan Probe and SYBR green assays. This was to explore the possibility that the apparently homozygous patterns of sequence variants in these patients and the apparently wild type patterns in their fathers would represent exon deletions on the paternal alleles [10]. Assays were designed for exon 2 of the *IDUA* gene containing the apparently homozygous variant of patient A; exons 1 and 3–14 served as control exons. Assays of the *GAA* gene concerned exon 13 containing the apparently homozygous variant of patient B; exons 3, 8 and 18 served as control exons. All assays gave normal results for patients A and B and their parents (results not shown). This indicates that heterozygous partial gene deletions were not the cause of the observed discrepancies in these patients’ and paternal DNAs.

### Analyses of microsatellite markers and microarrays

**Fig. 2:**
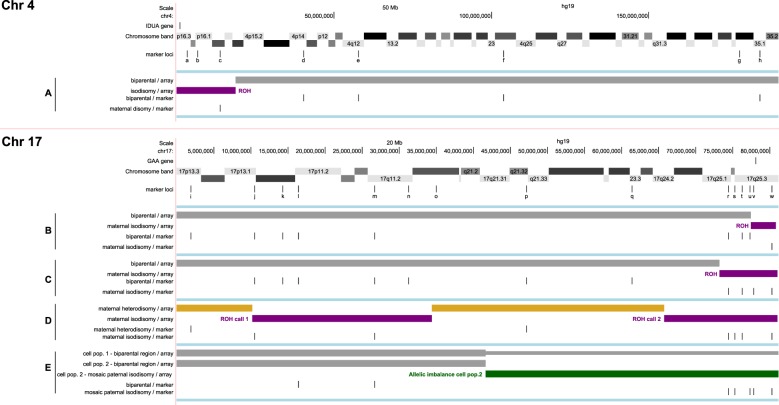
Graphic images of chromosome 4 (patient A) and chromosome 17 (patients B–E), based on the results of microsatellite marker and SNP genotyping array analyses. The microsatellite marker loci are listed in Table 1. The colours of the bars indicate: Gray – biparental contributions / absence of UPD. Purple – region of UPiD. Yellow – region of UPhD. Green - allelic imbalance, interpreted as admixture of biparental UPiD cell populations. Supplementary figures 1–5 contain array data

The parental contributions to the chromosomal areas involved were further investigated. For the *IDUA* gene, 8 microsatellite markers on chromosome 4 were tested, designated a-h in Fig. 2 and listed in Table 1. Of these, D4S412/a, D4S2935/b, D4S403/c and D4S405/d were located proximal to *IDUA* on the short arm and D4S1592/e, D4S1572/f, D4S415/g and D4S1535/h on the long arm of the chromosome. Four markers (D4S405/d, D4S1592/e, D4S1572/f and D4S1535/h) showed biparental inheritance (paternal contributions) in proband A’s DNA, three markers (D4S412/a, D4S2935/b and D4S415/g) were not informative, but for D4S403/c at 4p16.1 patient A showed absence of a paternal allele. Together with the apparently homozygous maternal sequence variant this suggested segmental uniparental isodisomy (sUPiD). To examine this further, SNP genotyping array analyses were performed.

For patient A, investigated on Affymetrix SNP6.0, the GTC software showed one region of homozygosity with a size of 18 Mb on chromosome 4pter (Fig. 2A; supplementary figure S1A and S1B). The homozygous, copy neutral, region comprised the *IDUA* gene and marker D4S403, which showed only a maternal contribution in the marker analysis.

For the cases involving the *GAA* gene (chromosome 17q25.2–25.3), 15 markers were tested including 6 markers at chromosome 17q25 (Table 1; designated i-w in Fig. 2). Of the latter, D17S785/r, D17S1790/s, D17S802/t, D17S836/u and D17S784/v were proximal to *GAA* and D17S928/w was distal to the gene (Fig. 2B–E). The remaining markers i-q covered 17p13.3 through 17q23.3. In DNA of proband B, the distal marker D17S1928/w showed one of the maternal alleles and no paternal allele. Markers D17S836/u, D17S802/t and D17S785/r at chromosome 17q25 showed biparental inheritance, as did the other informative markers at 17q11.2–17p13.3. Using array analysis *(*Affymetrix SNP6.0*)*, patient B showed one 3.5 Mb region of homozygosity on chromosome 17qter (Fig. 2B; supplementary figure S2A and S2B). This copy neutral region included the GAA gene and one of the marker loci, i.e. D17S928/w, that showed only the maternal allele in the marker analysis. The remaining chromosomal regions consisted of biparental contributions as evidenced by both normal array results and the informative markers. In DNA of patient C, markers D17S928/w, D17S784/v, D17S802/t and D17S785/r located on either side of the gene at 17q25 showed patterns in agreement with maternal UPiD (Fig. 2C, and supplementary figure S3). Seven markers throughout the remaining part of the chromosome showed biparental inheritance. Patient C was further analysed using the Illumina CytoSNP-12 platform. One region of (copy neutral) homozygosity of 8 Mb was observed encompassing the distal marker D17S928/w and *GAA* and ending just centromeric to the informative marker D17S785/r. The remaining chromosomal patterns were of biparental origin. We conclude that patients A, B and C were all cases of maternal sUPiD. For patient D (Fig. 2D; supplementary figure S4) all markers at 17q25 showed patterns in agreement with maternal UPD, of which D17S928/w, D17S802/t, D17S1790/s and D17S785/r were informative for UPiD. D17S1868/p at 17q21.32, however, showed maternal heterodisomy (UPhD), as did D17S831/i at 17p13.3, with 2 markers in between showing isodisomic patterns. Array analyses (Illumina CytoSNP-850K) showed allelic imbalance throughout the chromosome with ROH blocks at 17q25 and 17q12–17p13.1 coinciding with the regions of UPiD indicated by the markers, whereas the remaining areas were in agreement with maternal UPhD as they did not show ROH and lacked paternal contributions. These results indicate the presence of maternal whole chromosome UPD, with alternating regions of homozygosity and heterozygosity, consistent with a meiosis I error resulting in maternal UPhD.

Array analysis (Illumina CytoSNP-850K) was the most informative tool for patient E. Allelic imbalance of a large region (39.5 Mb) was detected, compatible with a segmental mosaic ROH (Fig. 2E; supplementary figure S5), as evidenced by an estimated 0.17 BAF deviation. His DNA showed biparental marker patterns throughout the chromosome. However, in agreement with the results of the mutation analysis, at the 17q25 region 5 of the markers had enhanced peaks of one paternal allele. We conclude that this case represents a mosaic paternal sUPiD.

## Discussion

Using microsatellite markers and SNP microarrays we identified 5 cases of UPD in patients with autosomal recessive lysosomal storage diseases, 4 of which were maternal in origin and 1 was paternal. It concerned one case of Hurler disease and four with Pompe disease including one case with an unusual combination of features. Four of these cases were traced when apparent homozygosity for a Hurler- or Pompe disease-causing variant was found in the patients and only the mothers proved to be carriers. Confirmation of biological parenthood was done by separate assay and/or was inherent in the results of the (comparative) analyses. Exon deletions and SNPs in primer areas had been excluded in patients A and B. For the patients we encountered more recently, microarray analyses became tools at an earlier stage of diagnostic work up, which enabled direct allelic quantification. Only one case (patient D) showed wUPD, with alternating regions of UPiD and UPhD reflecting a meiosis I origin [11]. The remaining cases represented postzygotic events resulting in segmental UPiDs, one of them in a visibly mosaic form and of paternal origin (patient E). This finding was particularly relevant for the diagnosis of patient E. He had presented with infantile cardiomyopathy, but has no muscle weakness, also as a toddler, which is an unusual combination. He had a decreased acid α-glucosidase activity in leukocytes that was *above* the range of known Pompe disease patients. However, the enzyme activity in cultured fibroblasts was within the patient range. Genotyping showed normal patterns in the mother’s DNA and heterozygosity for a known pathogenic variant in the father. In the patient both leukocytes and fibroblasts showed skewed heterozygous pattern with a more pronounced (paternal) *variant* allele than the (maternal) normal allele in the *fibroblast* DNA (Fig. 1). This may reflect the difference in enzyme activity between both tissues and strongly indicated mosaic sUPiD. Marker analyses on DNA from leukocytes and fibroblasts of this patient were less informative than in the other - non-mosaic - cases, where *absence* of parental contribution can be seen. Five markers did show enhanced peaks of one paternal allele, but the difference between the two tissues was less obvious. SNP genotyping array analysis on leukocyte DNA was clearly consistent with mosaicism, as 2 cell populations were visible (Fig. 2E; supplementary figure S5), while copy number neutral [12]. One may speculate that the resulting enzyme activity in skeletal muscle was above the threshold for clinical expression at the present age of the patient, whereas the activity in the heart was not sufficient to prevent cardiomyopathy.

The estimates of the occurrence of (germline) UPD vary considerably. The most widely cited prevalence of UPD-for-any-chromosome is 1/3500 births [13]. Using exome sequencing and microarray analysis King et al. [14] detected six cases of UPD, one of them segmental, in 1057 previously investigated but undiagnosed children with developmental disorders, which would represent a significant enrichment. Segmental UPiD has been attributed to postzygotic somatic recombination between maternal and paternal homologues or chromosomal breakage and repair using the homologous chromosome as a template [3, 15]. There is a tenfold difference between the reported numbers of wUPD (n = 3653) and sUPD (n = 363) [16]. It is conceivable that the number of reported sUPD will increase due to a more widespread application of high resolution analyses of the genome. The vast majority of reported sUPiD to date concerned paternal sUPiD of chromosome 11p causing Beckwith Wiedemann syndrome. Recently, mosaic sUPiD and progressive clonal selection have been identified as a common mechanism leading to late onset ß-thalassemia major [17].

After Sanger sequencing of 40 different AR genes in their clinical service, Landsverk et al. [18] reported 75 cases of apparent homozygosity with available parental DNAs. In 9/75 (12%) cases parental carrier status could not be confirmed. Four cases were attributed to deletions or allele drop out due to private SNPs in primer regions. In two cases UPiD was identified, one case each of segmental and complete UPD. However, UPiD was not unlikely in the three unresolved, but only partially analysed cases, which would bring the maximum percentage of UPD cases to 6.7%. In our diagnostic laboratory, Pompe disease is the most frequently tested lysosomal storage disease. In our files (1995-October 2015) were 272 index cases -of 32 nationalities- confirmed by *GAA* genotyping, 64 of whom were apparently homozygous for a pathogenic variant. In 35 of these cases both parents could be examined and we identified one case of total maternal UPD and two cases of segmental maternal UPiD including the *GAA* gene at 17q25.3 without evidence of mosaicism. In addition, one case of skewed *hetero*zygosity was shown to represent mosaic segmental paternal UPiD. Hence, we saw four cases (11%) of UPD among 36 cases where segregation could be tested. This percentage of cases that ‘*did not inherit the mutation from each parent*’ is similar to Landverk’s data, but we identified UPDs only and no cases with deletions or allele drop out.

The patients with sUPDs at 17q came from 4 different countries and their disomic regions were all different in size varying from 3.5 to 39.5 Mb, the latter covering about three quarters of chromosome 17q. This illustrates that the centromeric boundaries of the sUPDs are not confined to specific regions.

The number of Hurler/Scheie cases in our lab is lower than the number of Pompe cases. We tested 74 index patients, of whom 29 were homozygous for a pathogenic *IDUA* variant. We identified the case of sUPiD described in this report out of a total of 18 homozygous patients where both parents could be tested.

Since we argued that the occurrence of UPD may be underestimated due to unavailability of parental DNA, we compared the results with a cohort of cystic fibrosis (CF) patients tested in our lab with both parental DNAs available. In 120 homozygous cases segregation was consistent with AR inheritance. The occurrence of human disease resulting from UPD was first demonstrated already in 1988 in a CF patient with maternal UPiD for chromosome 7 by Spence et al. [19]. However, we did not identify any case of UPiD in a CF cohort of 120 patients. In the literature, we could find no more than 9 CF patients reported with (total and segmental) UPiD [20, 21]. This number seems low for a genetic disease with a mean calculated incidence of 1:3500 in Europe, based on data from 26 regional and national CF newborn screening programs [22]. Some underreporting in the literature cannot be excluded. Also, large numbers of patients may be needed for CF, where the relative contribution of UPD to the incidence of the disease may be lower due to the high carrier frequency. We propose to do a survey among diagnostic laboratories about their UPD cases to collect sufficient data for statistical analysis and investigate the frequency of UPD as a cause of AR disease.

Our results underline the clinical relevance of testing parental DNAs in the course of a routine diagnostic workup for DNA diagnostics of autosomal recessive diseases even when the parents are consanguineous (patient B). It is of direct importance to the parents in the first place, as a recessive disease caused by this mechanism has a negligible recurrence risk compared with the 25% that is conferred by biparental carriership.

Awareness of this genetic mechanism in diagnostic laboratories will speed up genotyping, when apparently conflicting results are found between the results of patients and parents. This is of particular relevance for patients with lysosomal storage diseases as a growing number of enzyme replacement programs become available where genotyping is a requirement for inclusion.

The case with the mosaic segmental UPD is very relevant with respect to cases with mild phenotypes (resembling those) of known recessive diseases. In the case of autosomal recessive diseases de novo sequence changes affecting gene (product) function appear to occur extremely rarely. Hence possible mosaicism can easily be overlooked or even ignored. When, however, genotypic or metabolic/enzymatic features are observed to vary according to the tissue analysed, mosaicism for segmental UPD must be considered.

Our observations also bear relevance for the protocol used for diagnostic workup for patients with developmental delay and intellectual disability that often involves SNP genotyping arrays at an early stage. It is important not only to check for genes involved in autosomal recessive diseases in “whole chromosome ROH” or repeated ROHs on one chromosome with interspersed heterozygosity indicating (whole chromosome) UPD. Even relative small ROHs (< 10 Mb) that include *telomeric* regions might indicate *segmental* UPD (e.g. patients B and C). We recommend that diagnostic laboratories report terminal ROHs regardless of size or the imprinting status of the chromosome.

## Supplementary information

LEGENDS TO SUPPLEMENTARY FIGURES

Supplementary figure S1A

Supplementary figure S1B

Supplementary figure S2A

Supplementary figure S2B

Supplementary figure S3

Supplementary figure S4

Supplementary figure S5
