# Supplementary material for: Segmental and total uniparental isodisomy (UPiD) as a disease mechanism in autosomal recessive lysosomal disorders: evidence from SNP arrays
Source: Eur J Hum Genet. 2019 Feb 8;27(6):919–27. doi: 10.1038/s41431-019-0348-y (PMC6777471; doi:10.1038/s41431-019-0348-y)
Supplement: Supplementary file 1 — LEGENDS TO SUPPLEMENTARY FIGURES [file 41431_2019_348_MOESM1_ESM.docx]

**Labrijn-Marks et al.**

**LEGENDS TO SUPPLEMENTARY FIGURES**

**Supplementary figure S1A.** Affymetrix SNP 6.0 array results for patient A, viewed in the Affymetrix Genotyping Console Browser. Within the same view, the informative markers and the genes of interest are displayed. The Region Of Homozygosity as well as the informative marker and genes of interest are indicated by means of callouts. From top to bottom: the LOH analysis result (blue bar), the probe signal (the blue dots represent individual Log2 Ratio values), the Copy Number analysis result (horizontal blue line, composed of dots), the informative microsatellite marker (vertical grey line), the RefSeq Gene annotations (green) including a callout for the location of IDUA and the ideogram of the shown chromosomal region.

**Supplementary figure S1B.** Affymetrix SNP 6.0 array results for patient A, viewed in Biodiscovery Nexus 7.5 software. From top to bottom: the chromosome 4 ideogram (IDUA in 4p16.3), the probe signal (grey dots representing individual probe Log2 Ratio values, range ±3, horizontal black lines indicate segmented regions), heterozygosity signal (grey dots representing individual BAF values, range 0 – 1, horizontal black lines indicate segmented regions) and LOH analysis positive results (yellow shaded segments).

**Supplementary figure S2A.** Affymetrix SNP 6.0 array results for patient B, viewed in the Affymetrix Genotyping Console Browser. Within the same view, the informative markers and the genes of interest are displayed. The Region Of Homozygosity as well as the informative marker and genes of interest are indicated by means of callouts. From top to bottom: the LOH analysis result (blue bar), the probe signal (the blue dots represent individual Log2 Ratio values), the Copy Number analysis result (horizontal blue line, composed of dots), the informative microsatellite marker (vertical grey line), the RefSeq Gene annotations (green) including a callout for the location of GAA, relative to the ideogram of the shown chromosomal region.

**Supplementary figure S2B.** Affymetrix SNP 6.0 array results for patient B, viewed in Biodiscovery Nexus 7.5 software. From top to bottom: the chromosome 17 ideogram (GAA in 17q25.3), the probe signal (grey dots representing individual probe Log2 Ratio values, range ±3, horizontal black lines indicate segmented regions), heterozygosity signal (grey dots representing individual BAF values, range 0 – 1, horizontal black lines indicate segmented regions) and LOH analysis positive results (yellow shaded segments).

**Supplementary figure** **S3.** Illumina Infinium CytoSNP-12 300K array results for patient C, and IBS duo analysis, viewed in Biodiscovery Nexus 7.5 software.

From top to bottom: the chromosome 17 ideogram (including location *GAA*), the probe signal (grey dots representing individual probe Log2 Ratio values, range ±1.5, horizontal black lines indicate segmented regions), heterozygosity signal (grey dots representing individual BAF values, range 0 – 1, horizontal black lines indicate segmented regions) and LOH analysis positive results (yellow shaded segments), IBS duo analysis of index vs Father, Mother respectively (grey dots representing individual genotype inheritance match (scoring 2,1,0)) with score 0 indicating complete mismatch of A/B genotyped individuals, and multiple co-localised 0 scores indicating absence of a paternal chromosome segment, explainable by sUPiD of maternal origin.

**Supplementary figure S4.** Illumina Infinium CytoSNP-850K array results for patient D, and IBS duo analysis, viewed in Biodiscovery Nexus 7.5 software.

From top to bottom: the chromosome 17 ideogram (including location *GAA*), the probe signal (grey dots representing individual probe Log2 Ratio values, range ±1.5, horizontal black lines indicate segmented regions), heterozygosity signal (grey dots representing individual BAF values, range 0 – 1, horizontal black lines indicate segmented regions) and LOH analysis positive results (yellow shaded segments), IBS duo analysis of index vs Father, resp. Mother (grey dots representing individual genotype inheritance match (scoring 2,1,0)) with score 0 indicating complete mismatch of A/B genotyped individuals, with chromosome wide 0 scores indicating absence of a paternal chromosome, explainable by whole UPD (wUPD) with alternating regions of maternal UPiD and UPhD.

**Supplementary figure S5.** Illumina Infinium CytoSNP-850K array results for patient E, viewed in Biodiscovery Nexus 7.5 software.

From top to bottom: the chromosome 17 ideogram, the probe signal (grey dots representing individual probe Log2 Ratio values, horizontal black lines indicate segmented regions), heterozygosity signal (grey dots representing individual BAF values, horizontal black lines indicate segmented regions) and LOH analysis positive results (yellow shaded segments)
