# Supplementary figures and images for: Segmental and total uniparental isodisomy (UPiD) as a disease mechanism in autosomal recessive lysosomal disorders: evidence from SNP arrays
Source: Eur J Hum Genet. 2019 Feb 8;27(6):919–27. doi: 10.1038/s41431-019-0348-y (PMC6777471; doi:10.1038/s41431-019-0348-y)

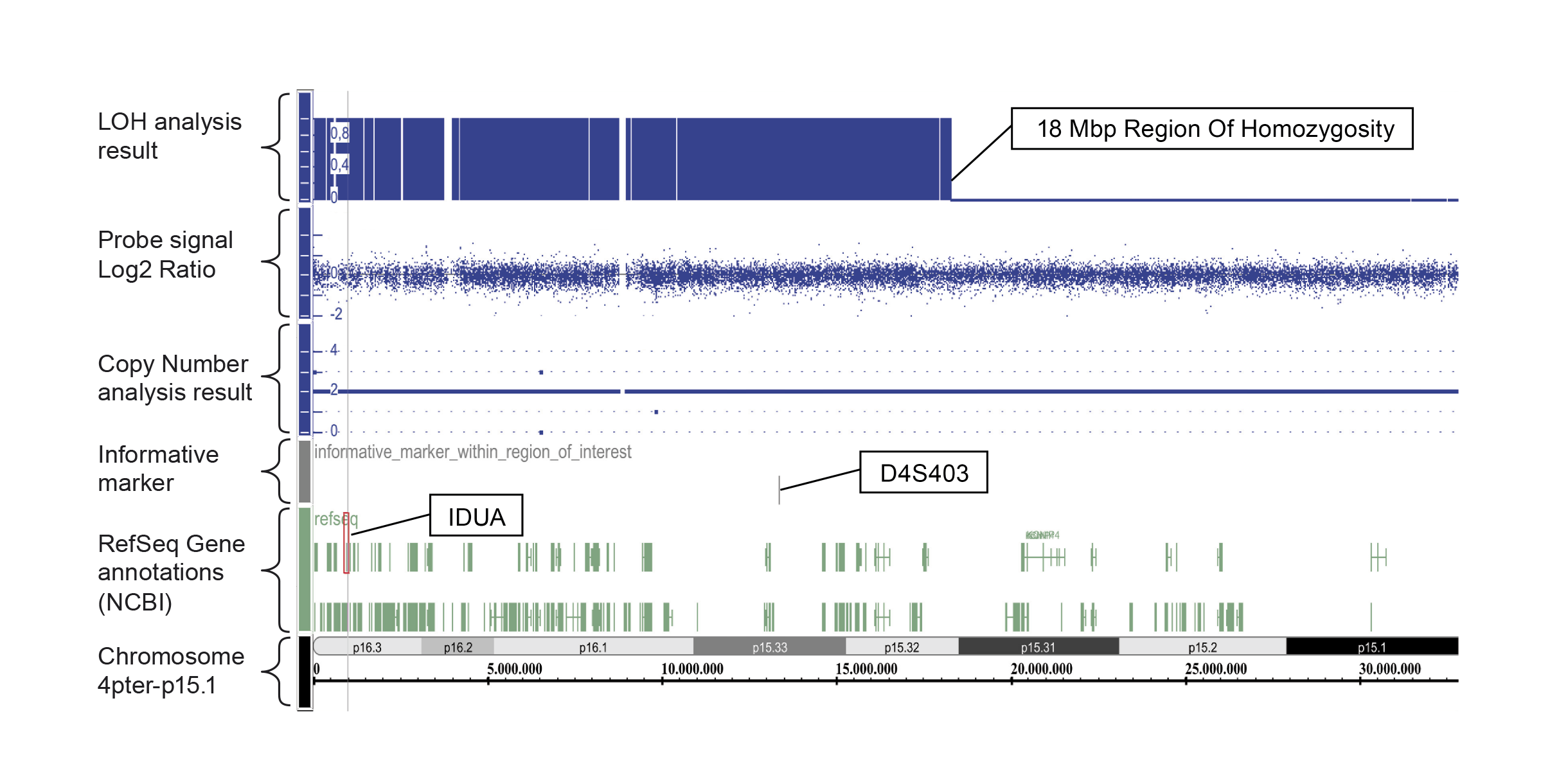

Supplement: Supplementary file 2 — Supplementary figure S1A [file 41431_2019_348_MOESM2_ESM.tif]

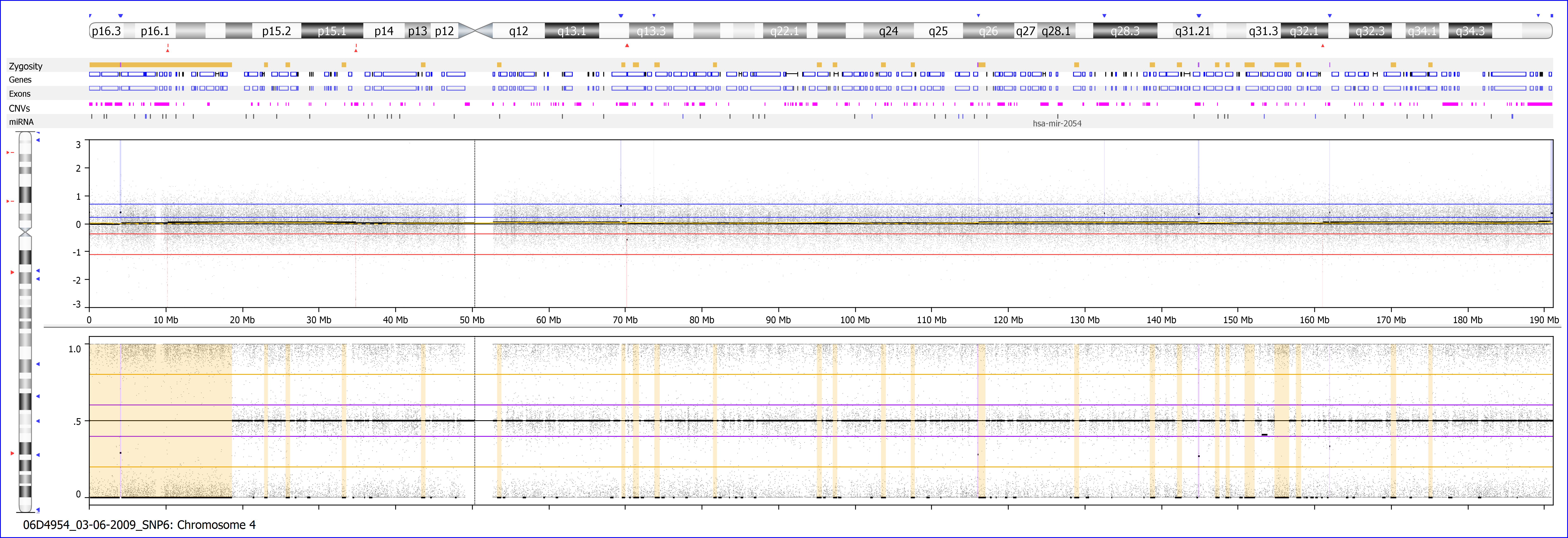

Supplement: Supplementary file 3 — Supplementary figure S1B [file 41431_2019_348_MOESM3_ESM.tif]

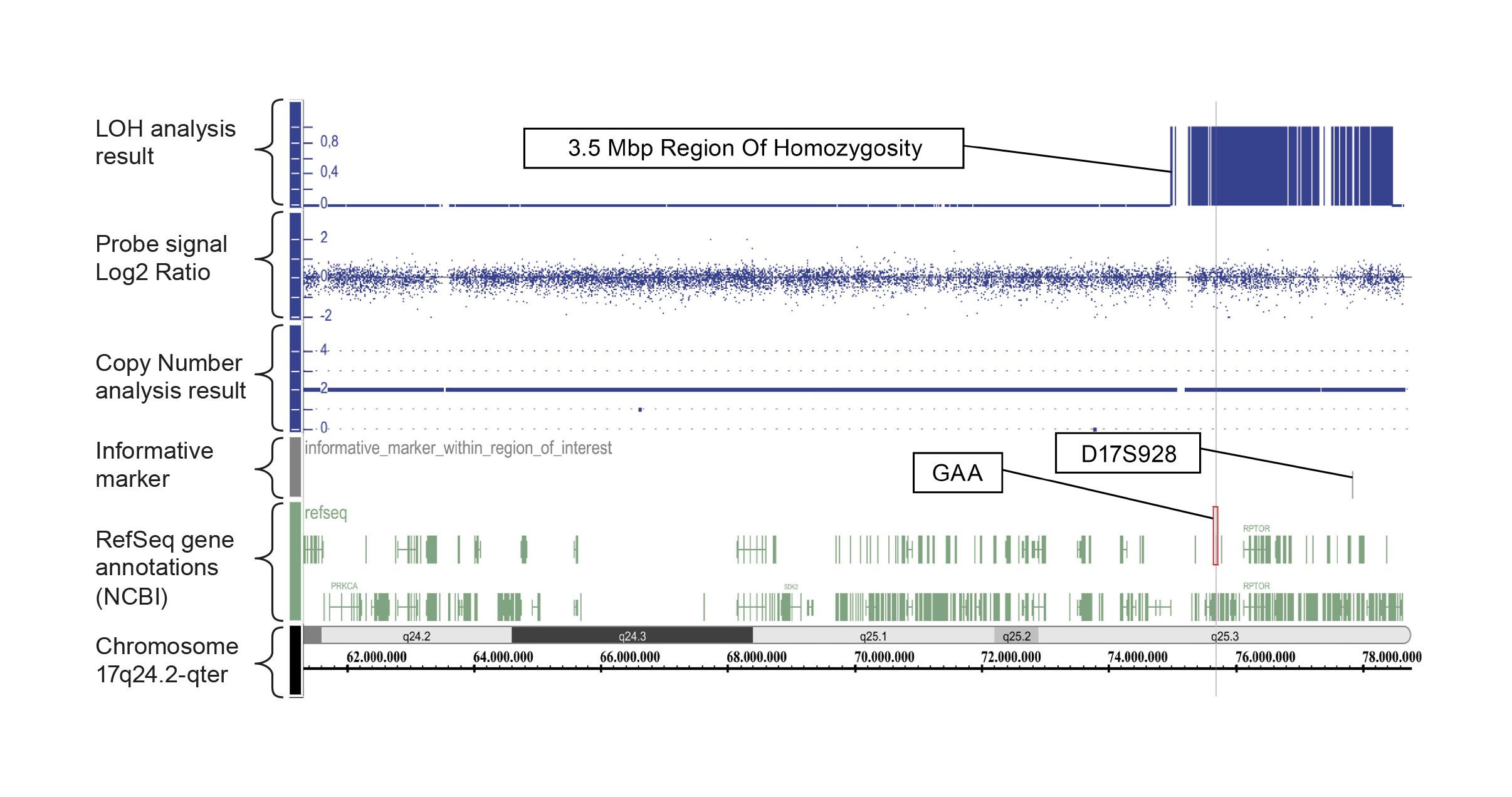

Supplement: Supplementary file 4 — Supplementary figure S2A [file 41431_2019_348_MOESM4_ESM.tif]

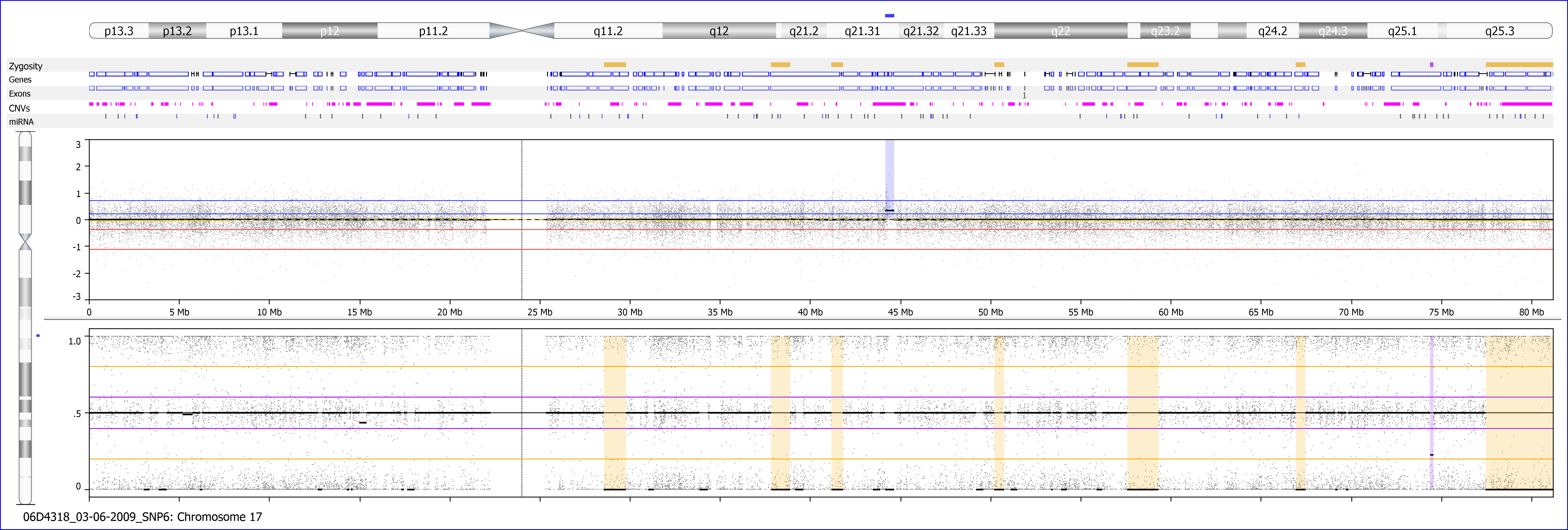

Supplement: Supplementary file 5 — Supplementary figure S2B [file 41431_2019_348_MOESM5_ESM.tif]

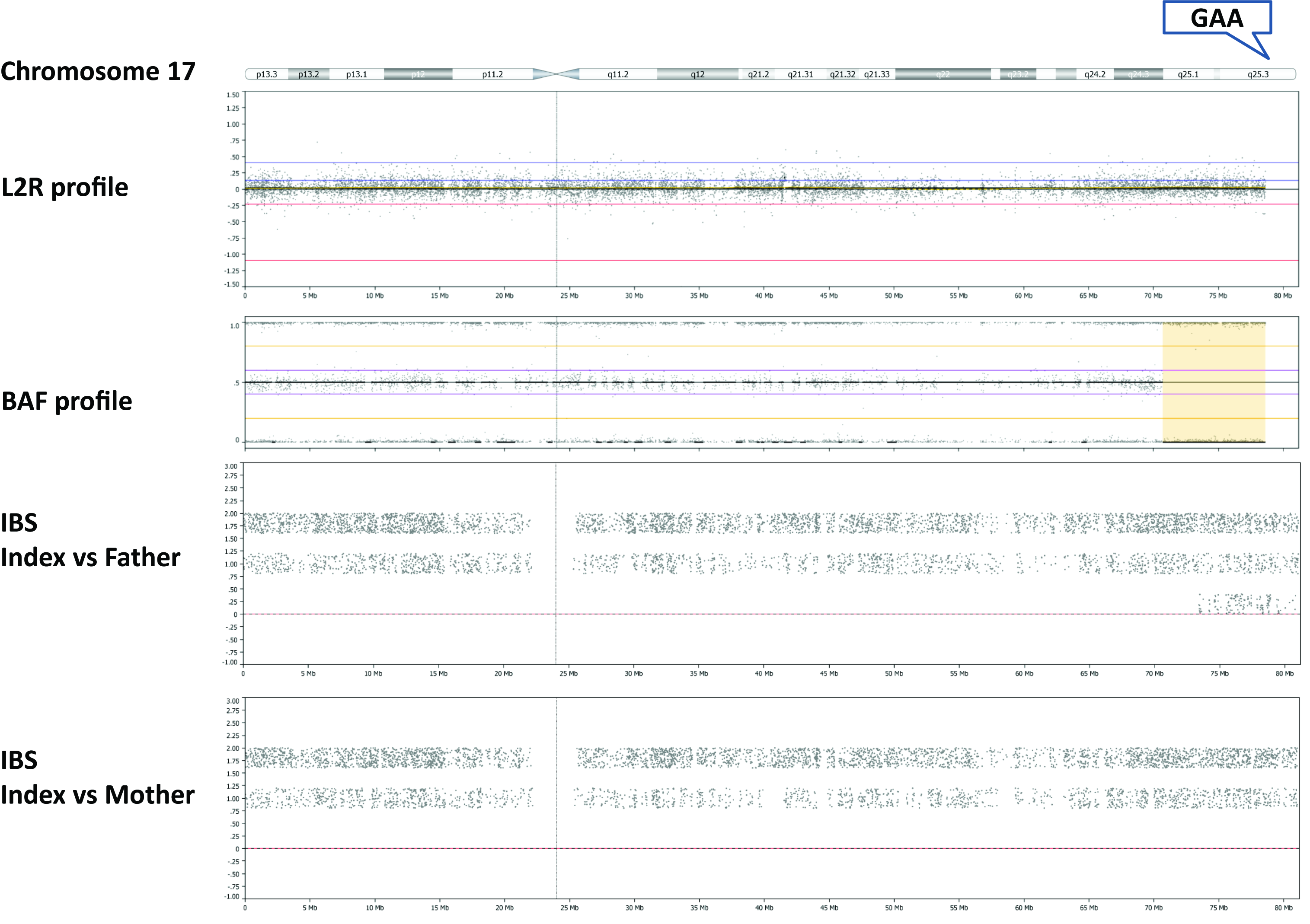

Supplement: Supplementary file 6 — Supplementary figure S3 [file 41431_2019_348_MOESM6_ESM.tif]

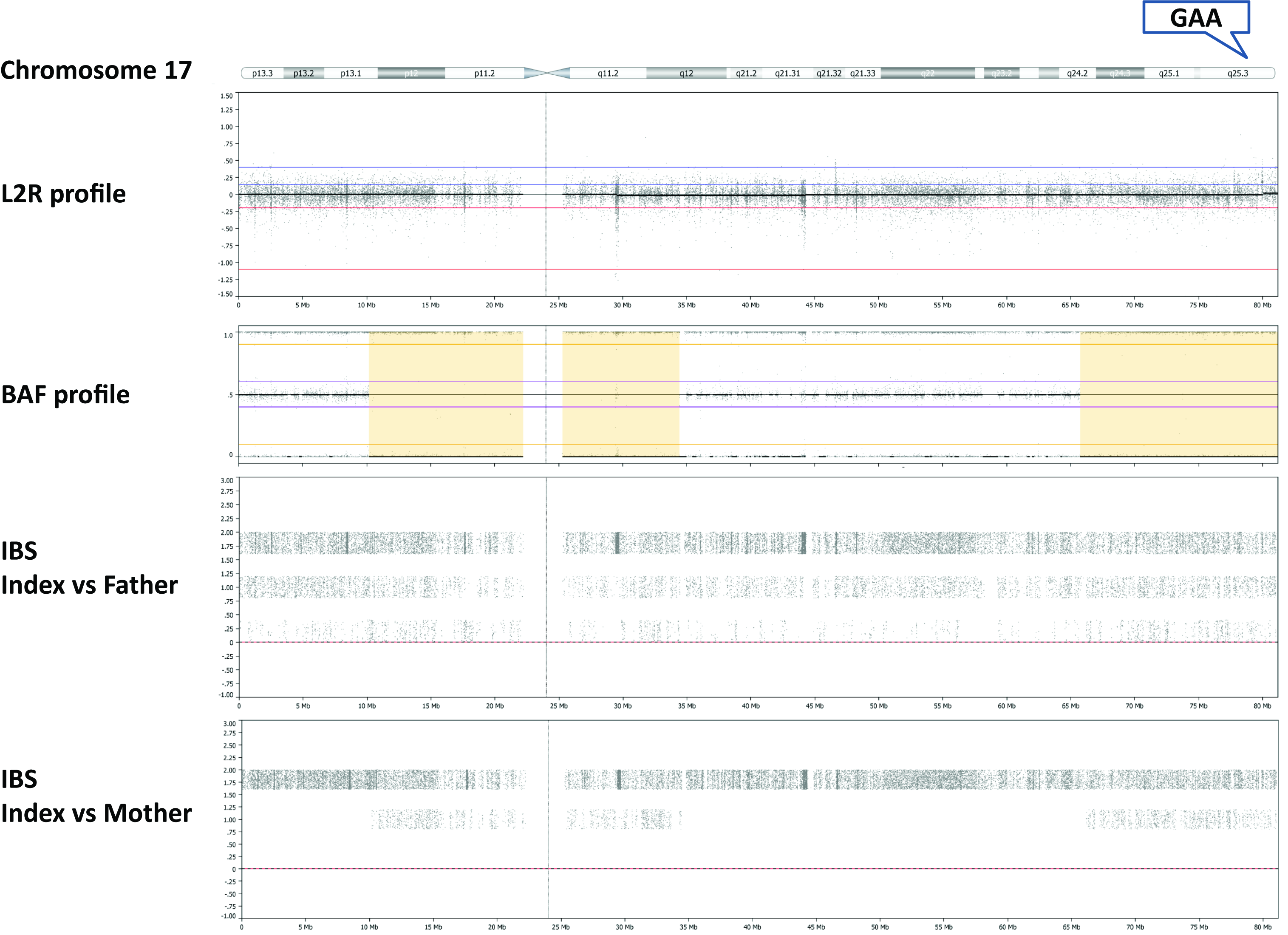

Supplement: Supplementary file 7 — Supplementary figure S4 [file 41431_2019_348_MOESM7_ESM.tif]

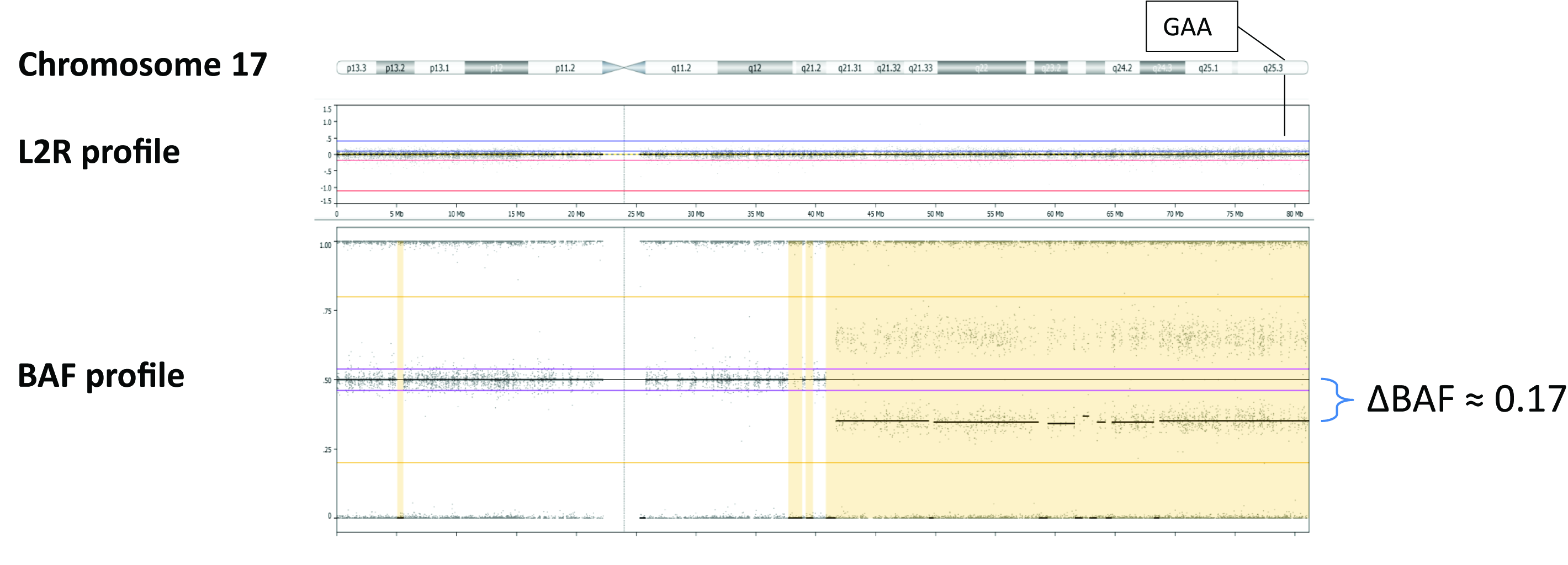

Supplement: Supplementary file 8 — Supplementary figure S5 [file 41431_2019_348_MOESM8_ESM.tif]
